# A Prognostic Model of Bladder Cancer Based on Metabolism-Related Long Non-Coding RNAs

**DOI:** 10.3389/fonc.2022.833763

**Published:** 2022-02-25

**Authors:** Jintao Hu, Cong Lai, Zefeng Shen, Hao Yu, Junyi Lin, Weibin Xie, Huabin Su, Jianqiu Kong, Jinli Han

**Affiliations:** ^1^ Department of Urology, Sun Yat-sen Memorial Hospital, Sun Yat-sen University, Guangzhou, China; ^2^ Guangdong Provincial Clinical Research Center for Urological Diseases, Sun Yat-sen Memorial Hospital, Sun Yat-sen University, Guangzhou, China

**Keywords:** bladder cancer, TCGA, metabolism, lncRNA, nomogram, prognosis

## Abstract

**Background:**

Some studies have revealed a close relationship between metabolism-related genes and the prognosis of bladder cancer. However, the relationship between metabolism-related long non-coding RNAs (lncRNA) regulating the expression of genetic material and bladder cancer is still blank. From this, we developed and validated a prognostic model based on metabolism-associated lncRNA to analyze the prognosis of bladder cancer.

**Methods:**

Gene expression, lncRNA sequencing data, and related clinical information were extracted from The Cancer Genome Atlas (TCGA). And we downloaded metabolism-related gene sets from the human metabolism database. Differential expression analysis is used to screen differentially expressed metabolism-related genes and lncRNAs between tumors and paracancer tissues. We then obtained metabolism-related lncRNAs associated with prognosis by correlational analyses, univariate Cox analysis, and logistic least absolute shrinkage and selection operator (LASSO) regression. A risk scoring model is constructed based on the regression coefficient corresponding to lncRNA calculated by multivariate Cox analysis. According to the median risk score, patients were divided into a high-risk group and a low-risk group. Then, we developed and evaluated a nomogram including risk scores and Clinical baseline data to predict the prognosis. Furthermore, we performed gene-set enrichment analysis (GSEA) to explore the role of these metabolism-related lncRNAs in the prognosis of bladder cancer.

**Results:**

By analyzing the extracted data, our research screened out 12 metabolism-related lncRNAs. There are significant differences in survival between high and low-risk groups divided by the median risk scoring model, and the low-risk group has a more favorable prognosis than the high-risk group. Univariate and multivariate Cox regression analysis showed that the risk score was closely related to the prognosis of bladder cancer. Then we established a nomogram based on multivariate analysis. After evaluation, the modified model has good predictive efficiency and clinical application value. Furthermore, the GSEA showed that these lncRNAs affected bladder cancer prognosis through multiple links.

**Conclusions:**

A predictive model was established and validated based on 12 metabolism-related lncRNAs and clinical information, and we found these lncRNA affected bladder cancer prognosis through multiple links.

## Introduction

Bladder cancer, as a common tumor of the urinary system, has always been the focus of research. Recent studies have shown that the metabolism of glycogen, lipid, amino acid, and other substances is closely related to the diagnosis and prognosis of tumors (1, 2). For example, in cancer cells, blocking the supply of lipids can have serious effects on bioenergetics, membrane biosynthesis, and intracellular signaling processes (3). And the specificity of glycogen metabolism in tumor cells, the Warburg effect, has also been widely studied and discussed (4–7). These studies have opened up potential therapeutic targets for tumors. And the tumor metabolism-based therapy treatment has led to improved cancer outcomes (2, 8). Despite the success of previous studies in improving the prognosis of bladder cancer, there were 213,000 deaths from bladder cancer worldwide in 2020 (9). And little reformation in treatment has occurred in recent years. Therefore, we still need to explore new biomarkers to aid in diagnosis and treatment.

The lncRNAs play a crucial role in transcriptional regulation, epigenetic gene regulation, and disease, a kind of non-coding RNAs greater than 200 nucleotides, with no protein-coding function (10, 11). With the deepening of research, the relationship between lncRNA and progression, metastasis, and prognosis of various tumors has been found, such as the digestive, nervous, bone, and urinary system (12–17).

For bladder cancer, immune-associated and autophagy-associated lncRNAs were found as a marker of early diagnostic and prognostic treatment (18–20). Recent studies show that lncRNAs play important roles in glucose and lipid (21, 22).

Although we know the nonnegligible fact that metabolism-related genes and lncRNAs are involved in tumor prognosis. At present, the research on metabolism-related lncRNA is not abundant, which is the starting point of this dissertation. It is of great underlying value to study metabolism-related lncRNA to accurately predict the prognosis of bladder cancer. Hence, to address the clinical values, we tried to establish a nomogram containing metabolism-related lncRNAs and clinical data and explore the possible functions of metabolism-related lncRNAs beings through the GSEA.

## Methods

### Data Collection and Processing

The RNA-Seq, lncRNA sequencing data, and related clinical information of bladder cancer were extracted from TCGA (https://portal.gdc.cancer.gov). And metabolism-related gene sets were downloaded from the GSEA database (https://www.gsea-msigdb.org/gsea/index.jsp). Taking | log2FC | > 0.5 and *P* < 0.05 as standards, the “edgeR” package was used to construct the volcano map and distinguish the differentially expressed metabolism-related gene and lncRNAs in tumors and paracancer tissues. The correlation between metabolism-related genes and lncRNAs is measured by the Pearson correlation coefficient. Metabolism-related gene and lncRNAs whose correlation coefficients satisfy | R2 | > 0.5 and *P* < 0.05 are considered related and used for further analysis.

### Identification of Prognostic Related LncRNAs and Construction and Validation Risk Score Model

Patients with a follow-up time no less than 30 days were divided into the training group and the validation group in a ratio of 2 to 1. Univariate Cox regression analysis was used to analyze clinical survival data and lncRNAs. These lncRNAs associated with prognosis were further screened by LASSO regression analysis. The n selected lncRNAs were analyzed by multivariate Cox regression analysis, and the risk scoring model was based on coefficients. The AUC was used to evaluate the scoring model.

We can calculate the risk score for each patient using the scoring model. The patients were divided into high and low-risk groups based on the median risk score. The Kaplan-Meier survival curve was plotted to show the difference in prognosis between the two groups. The risk score and survival status of each sample were presented using risk curves and scatter plots. Heat maps were used to show the expression state of selected lncRNAs.

### Construction and Aessement Nomogram

Risk scores, age, gender, and TNM were included in univariate and multivariate Cox analysis to obtain variables associated with prognosis. Then, a nomogram was established based on the results of multivariate Cox analysis. We evaluated the nomogram by calculating the C-index and plotting the area under the receiver operating characteristic curve (AUC), calibration plot, and decision curve analysis (DCA).

### Gene-Set Enrichment Analysis

The differentially expressed metabolism-related genes in the high-risk group and low-risk groups were analyzed for gene enrichment. To explore what biological functions or pathways these expressed different genes might be involved in.

### Statistical Analysis

All statistical computations were conducted using the R software, version 4.0.2 (The R Foundation for Statistical Computing, Vienna, Austria http://www.R-project.org). And *P* < 0.05 was considered statistically significant.

## Results

### Differentially Expressed Genes and LncRNAs

**Figure1:**
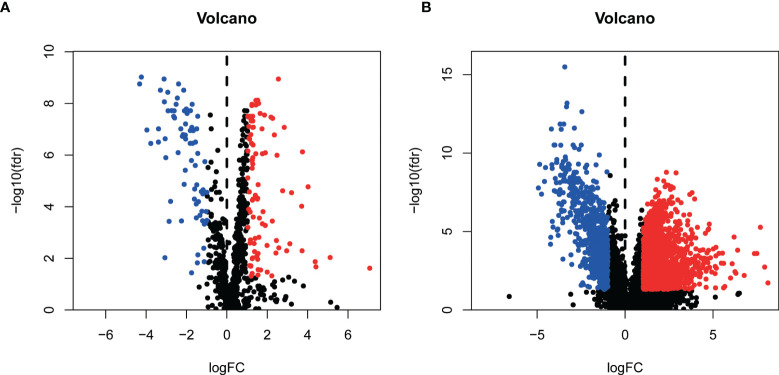
Identification of differentially expressed Metabolism-related genes [volcano plots **(A)**] and lncRNAs [volcano plots **(B)**].

We grabbed RNA-seqs, lncRNA sequences, and clinical data of bladder cancer tissues and paracancer tissues data from the TCGA database. And 937 metabolism-related gene sets were downloaded from the GSEA database. 169 differentially expressed metabolism-related genes **(** **Additional File: Table 1** **)** and 2301 differentially expressed lncRNAs **(** **Additional File: Table 2** **)** were obtained by differential expression analysis (**Figure 1**).

### Identification of Metabolism-Related LncRNAs Associated With Prognosis

**Figure 2:**
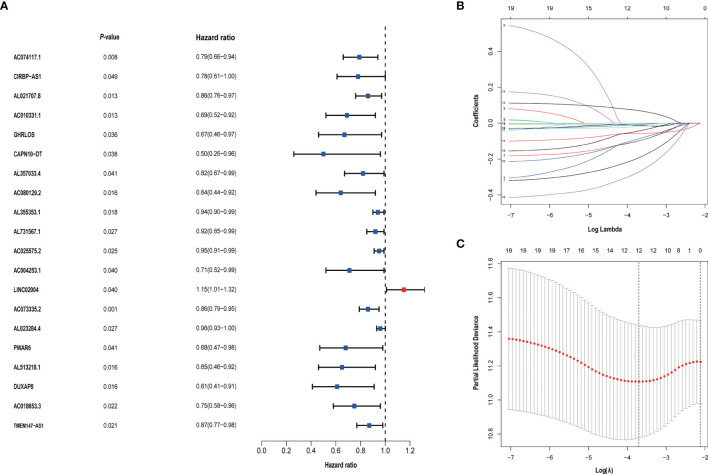
**(A)** Univariate Cox regression analysis was used to screen prognostic-related metabolism-related lncRNAs. **(B, C)** The screened 20 prognostic-related metabolism-related lncRNAs were incorporated into the Lasso regression model and penalties were used for controlling the overfitting effects of the model.

We identified 105 metabolism-related lncRNAs by correlation Pearson analysis, as shown in **Additional File Table 3**. And univariate Cox regression was performed, we screened out 20 metabolism-related lncRNAs associated with prognosis. To control overfitting, LASSO regression analysis was used, and finally, 12 metabolism-related lncRNAs associated with prognosis were obtained. These selected lncRNAs were CIRBP.AS1, AC018653.3, AL357033.4, LINC02004, DUXAP8, AC010331.1, PWAR6, AC025575.2, AL355353.1, AL731567.1, AC074117.1 and AC073335.2. Among them, LINC02004 is unfavorable to the prognosis of bladder cancer **(** **Figure 2** **)**.

### Construction and Validation Risk Score Model

393 patients were divided into training group (262) and validation group (131). In the training group, the risk score model was determined by multivariate cox regression analysis to calculate the coefficients for selected lncRNAs.


$$\text{Risk score}=\Sigma_{i=1}^{12}\;coefficient_{i}*EXP{\left(lncRNA\right)}_{i}$$

**Figure 3:**
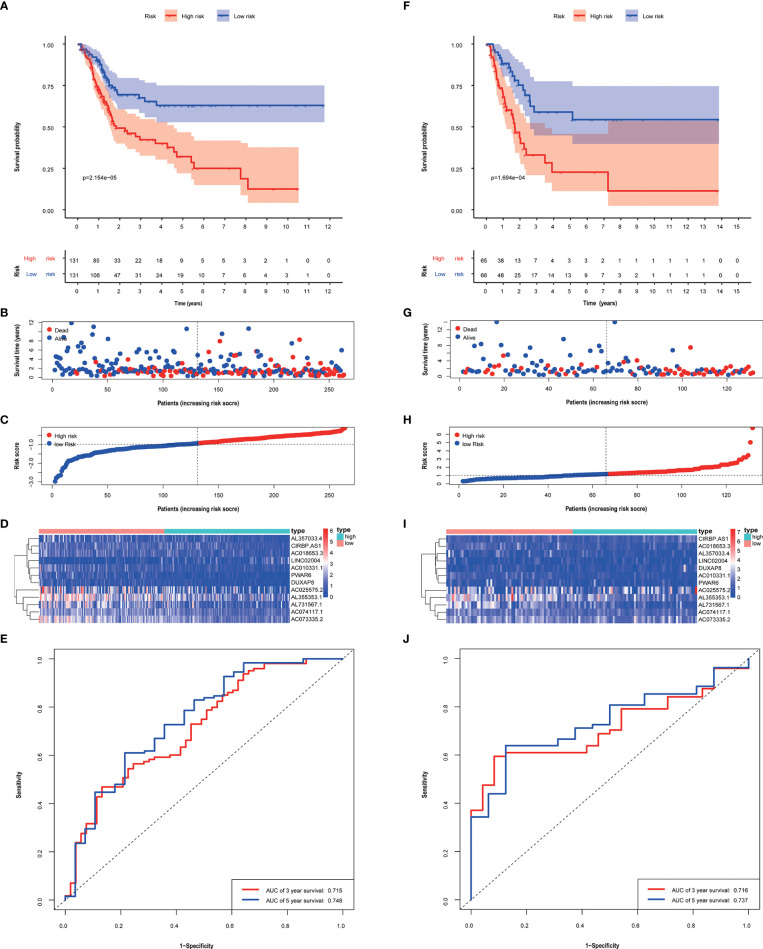
The performance of risk score model based on 12 metabolism-related lncRNAs in training **(A–E)** and validation groups **(F–J)**. Kaplan–Meier curves showed that patients with high-risk scores had a worse overall survival than patients in the low-risk group. **(A, F)** Patients with low-risk scores generally had longer survival years, and lower mortality than the other patients. **(B, C, G, H)** Heat maps of the risk scores were based on metabolism-related lncRNAs in the training group **(D)** and validation group **(I)**. ROC curves and AUC values of 3-, 5- year of risk score model in the training group **(E)** and validation group **(J)**.

The median risk score of patients calculated based on this risk score model was used to be divided into high and low-risk groups. In the training group and validation group, scores and gene expression, and survival prognosis for high and low risk are shown (**Figure 3**). The high-risk group had an unfavorable prognosis than the low-risk group (*P* < 0.05). And in both the training group and validation group, the AUC was greater than 0.71 (**Figure 3**). Furthermore, in **Additional File 1**, the good predictive effectiveness of the risk scoring model in each subgroup (age, sex, TNM stage) was presented.

### Construction and Aessement Nomogram

**Figure 4:**
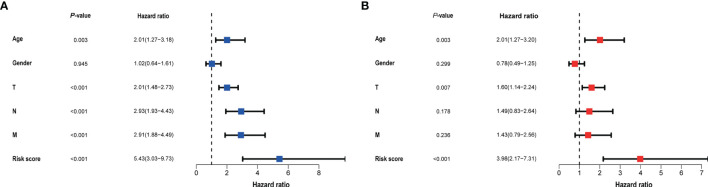
The result of univariate **(A)** and multivariate **(B)** Cox regression performed in the risk score and clinical data. Both in the two forests above suggested that the risk score was an independent prognostic factor.

**Figure 5:**
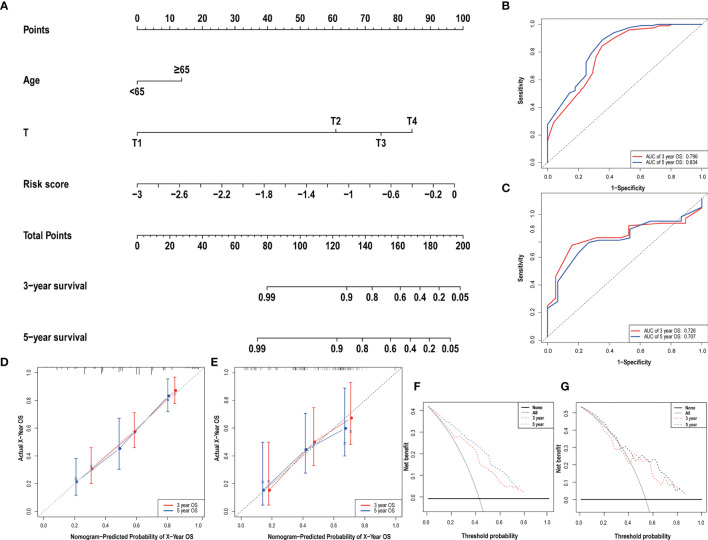
The nomogram **(A)** of the risk score model and its predictive power and reliability in the training group **(B, D, F)** and validation group **(C, E, G)**.

Clinical data and scores were included in the univariate and multivariate analysis (**Figure 4**). We constructed a nomogram based on the results of multivariate Cox analysis including sick score, age, and T stage. The c-index of the training group and validation group are 0.741 and 0.689 respectively. The ROC and DCA Curves showed satisfactory results (**Figure 5**). These results indicate that our nomogram is reliable and has clinical application value.

### Gene-Set Enrichment Analysis

**Figure 6:**
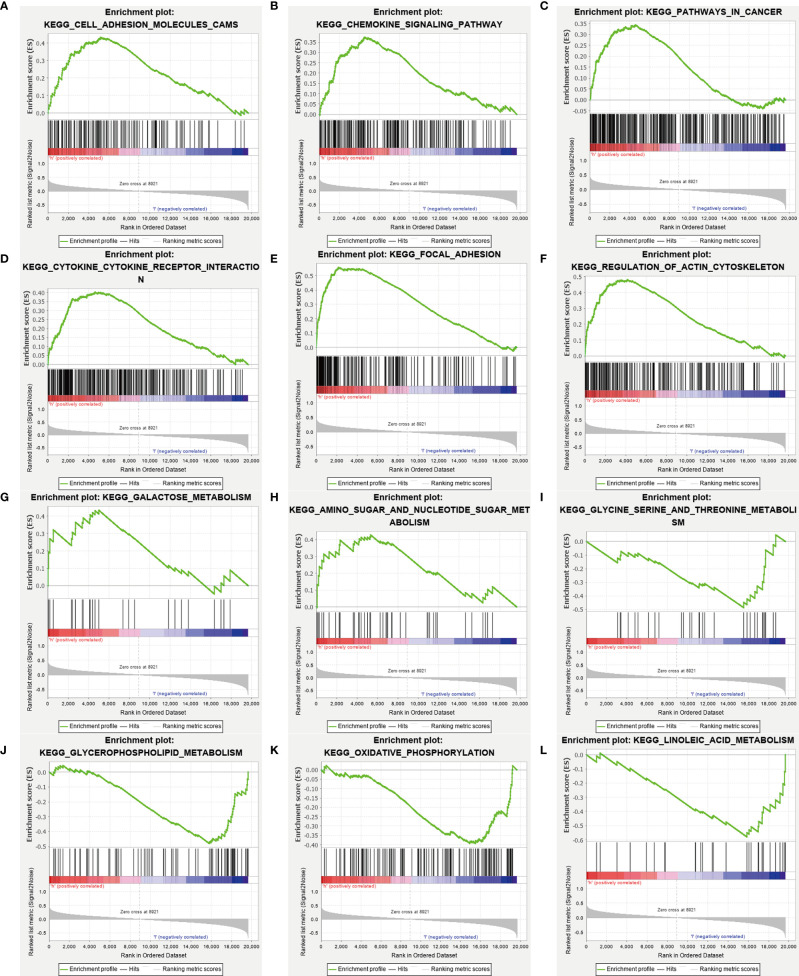
Gene-set enrichment analysis. The analysis results showed significant enrichment in the high-risk group **(A–H)**, and in the low-risk group **(I–L)**. And the metabolism-related genes are enriched in the high-risk group **(G, H),** and the low-risk group **(I–L)**.

We performed Gene Ontology (GO) functional annotation and Kyoto Encyclopedia of Genes and Genomes (KEGG) pathway enrichment analysis in differentially expressed metabolism-related genes between high and low-risk groups. Then we presented 9 KEGG molecular pathways in which different genes were enriched **(** **Figure 6**). Some metabolic pathways enriched into the high-risk group included galactose metabolism and sugar and nucleotide sugar metabolism. And glycine serine and threonine metabolism, glycerophospholipid metabolism, oxidative phosphorylation, and linoleic acid metabolism enriched into the low-risk group.

## Discussion

Worldwide, bladder cancer is characterized by high incidence, poor prognosis, and high economic burden. How to accurately predict prognosis and explore new therapeutic targets are urgently needed in the clinic. Previous studies have found metabolism-related genes and lncRNAs affect the prognosis of bladder cancer in different aspects (1, 23). However, there is little research on the relationship between metabolism-related lncRNAs and bladder cancer. This thesis fills the research gap by identifying metabolism-related lncRNAs associated with bladder cancer, exploring their function channels, and developing a nomogram to predict prognosis individually.

Although there are few studies on the prognostic relationship between metabolism-related related lncRNAs and bladder cancer, the prediction model based on other types of lncRNAs has a good performance in predicting the prognosis of bladder cancer. In tumor immunity, an 8 immune-related LncRNA classifier for prognostic prediction, could predict prognosis and immunotherapeutic response (24). And patients with bladder cancer grouped according to identified immune-related lncRNA signature showed different immune states (25, 26). Ferroptosis, an iron-dependent form of nonapoptotic cell death has been proved to be closely related to the development of cancer (27). The ferroptosis-related lncRNA signatures can predict the prognosis of bladder cancer patients, which still needs further experimental verification in the future (28).

From the Kaplan-Meier survival curve we drew according to subgroups, we could see that this model still has good predictive performance in most subgroups age > 65, female, male, T1-2, T3-4, N0, and M0. In the remaining three subgroups, no significant differences were observed, and the insufficient sample size was considered as the main reason.

Our study developed a risk score model with good reliability. This reconfirmed that these selected metabolism-related lncRNA (CIRBP.AS1, AC018653.3, AL357033.4, LINC02004, DUXAP8, AC010331.1, PWAR6, AC025575.2, AL355353.1, AL731567.1, AC074117.1 and AC073335.2.) are dysregulated in bladder cancer.

LncRNA DUXAP8 was located on chromosome 20q11 with 2307 bp in length8 (29). DUXAP8 can regulate PTEN to alter the prognosis of bladder cancer (30). Similar to Liu’s research, DUXAP8 might be a useful lncRNAs resource for prognostic or diagnostic markers for bladder cancer (31). Recent studies have found that silencing DUXAP8 expression can inhibit cell proliferation and promote apoptosis by targeting miR-26b-5P (32). DUXAP8 is involved in the initiation of many tumors and has the potential as a therapeutic target (33). LncRNA PWAR6 regulates proliferation and migration by epigenetically silencing YAP1 in tumorigenesis of pancreatic ductal adenocarcinoma (34). One study has adopted a machine-learning Algorithm and Laplacian score feature selection, and an advanced over-sampling, they found AC074117.1 is the target of multiple cancer-related miRNAs and interacts with adjacent protein-coding genes (35). RNA is associated with prognosis in metastatic lung Adenocarcinomaand rectal adenocarcinoma (36, 37). Also according to existing research findings, AC010331.1, a favorable prognostic factor, can improve prognosis by upregulating autophagy-related gene expression (38). AC018653.3 and CIRBP.AS1 may also work through regulating Ferroptosis-related genes and N6-methyladenosine related genes respectively (39, 40). These suggest that abnormal regulation of some lncRNA may affect multiple aspects of bladder tumors cell. The rest of the lncRNAs (AL357033.4, LINC02004, AC025575.2, AL355353.1, AL731567.1, and AC073335.2) have not been studied in bladder cancer yet. We believe these lncRNAs could be a new focus.

We found through GSEA that different metabolism-related pathways were significantly enriched both in high and low-risk groups. The galactose metabolism and amino sugar and nucleotide sugar metabolism enrich in patients with high-risk scores, which might mean a poor prognosis in these patients. Consistent with our research, Overexpression of the galactose transporter and galactose binding lectin, may contribute to tumor progression (41, 42). On the contrary, serine and threonine metabolism, glycerophospholipid metabolism, oxidative phosphorylation, and linoleic acid metabolism are enriched in patients who would have a favorable prognosis. The potential value of serine and threonine metabolism and linoleic acid metabolism as a therapeutic target has been demonstrated in some studies (43, 44).

We were the first to build a nomogram based on metabolism-related lncRNA. After verification and evaluation, the model can accurately predict the prognosis of bladder cancer. Using GSEA, we initial research the potential function of 12 metabolism-related lncRNA. Admittedly, there are also some limitations to our study. First, further experiments on cells and animals are needed to verify the function of this lncRNA. Second, contained clinical data lacks some important data, such as comorbidities, therapies, smoking, and the cause of death.

## Conclusion

We identified 12 metabolism-related lncRNAs associated with the prognosis of bladder cancer. And we found these lncRNA affecting bladder cancer prognosis through multiple links could be the focus of future research. From this, the first nomogram was established and validated based on metabolism-related lncRNAs and clinical information.

## Data Availability Statement

The original contributions presented in the study are included in the article/**Supplementary Material**. Further inquiries can be directed to the corresponding authors.

## Author Contributions

JTH, JLH, and JK conceived and designed the study, participated in the collection of data and data analysis, and drafted the manuscript. CL and ZS assisted in the design of this research and project development. HY, JL, WX, and HS analyzed the data and reviewed the article. All authors contributed to the article and approved the submitted version.

## Funding

This study was supported by the Science and Technology Planning Project of Guangdong Province (Grant No. 2020A1515111119) and Guangdong Provincial Clinical Research Center for Urological Diseases (Grant No.2020B1111170006). The funders played no role in design of the study, collection, analysis, and interpretation of data or in writing the manuscript.

## Conflict of Interest

The authors declare that the research was conducted in the absence of any commercial or financial relationships that could be construed as a potential conflict of interest.

## Publisher’s Note

All claims expressed in this article are solely those of the authors and do not necessarily represent those of their affiliated organizations, or those of the publisher, the editors and the reviewers. Any product that may be evaluated in this article, or claim that may be made by its manufacturer, is not guaranteed or endorsed by the publisher.

## Supplementary Material

Supplementary Table 1 169 metabolism-related genes differentially expressed in tumors and paracancer tissues.

Supplementary Table 2 2301 lncRNAs differentially expressed in tumors and paracancer tissues.

Supplementary Table 3 105 metabolism-related lncRNAs were identified.

Supplementary Figure 1 The survival outcomes of bladder cancer patients with different risk scores in subgroups. **(A)** Age < 65, **(B)** Age ≥ 65, **(C)** Female, **(D)** Male, **(E)** T1-2, **(F)** T3-4, **(G)** N0, **(H)** N1-3, **(I)** M0, **(J)** M1.
